# Fibrinogen as a Driver of Oxidative Stress and Apoptosis in the Fetal Brain After Maternal Influenza

**DOI:** 10.21203/rs.3.rs-10396951/v1

**Published:** 2026-07-21

**Authors:** Rafael J. Gonzalez-Ricon, Fernando J. Rigal, Izan Chalen, Adrienne M. Antonson

**Affiliations:** University of Illinois Urbana-Champaign; University of Illinois Urbana-Champaign; University of Illinois Urbana-Champaign; University of Illinois Urbana-Champaign

**Keywords:** Maternal immune activation, gestational influenza, fibrinogen, microglia, oxidative stress, SOX2 progenitors, apoptosis, sex-biased vulnerability

## Abstract

Maternal infection during pregnancy has been associated with increased risk for adverse neurodevelopmental outcomes, yet the mechanisms linking maternal inflammation to fetal brain vulnerability remain under active investigation. Here, we used a mouse-adapted gestational influenza A virus (IAV; X31) model of maternal immune activation to assess cerebrovascular leakage of fibrinogen, fibrinogen-microglia colocalization, oxidative stress-related responses, and cell death in fetal brains. Maternal IAV infection induced classic sickness symptoms and fetoplacental growth restriction, reflected by reduced fetal weight, crown-to-rump length, and placental weight. In the fetal brain, maternal flu increased fibrinogen accumulation in the subventricular zone (SVZ), while fibrinogen-microglia colocalization increased in the SVZ, thalamus-third ventricle interface, and whole hemisphere, suggesting broad fibrinogen-microglia proximity. These changes were accompanied by increased Iba1^+^/p47^phox+^ cell density in the same regions, consistent with greater microglial oxidative state. Because these regions contain neurogenic SOX2^+^ progenitors, we assessed whether fibrinogen accumulation and increased oxidation coincided with altered progenitor maintenance or survival. Although fetal SOX2^+^ neural progenitor density was not broadly altered by maternal flu, apoptosis analyses revealed a treatment- and sex-biased vulnerability, with males from IAV-infected dams demonstrating increased cell death, particularly within SOX2^+^ progenitors. Consistent with these *in vivo* findings, conditioned medium from fibrin-stimulated BV-2 microglia-like cells increased neural progenitor cell death *in vitro*, including amongst SOX2^+^ populations. Together, these findings support a framework in which maternal IAV-induced inflammation contributes to fibrinogen accumulation, microglial oxidative stress, and male-biased neural progenitor vulnerability in fetal brain regions critical for neurodevelopment.

## 1. Introduction

Influenza A virus (IAV; “flu”) infection during pregnancy has been associated with an increased risk of neurodevelopmental disorders (NDDs) in offspring, with schizophrenia (SZ) among the outcomes most consistently linked to maternal flu^1–5^. Although the worldwide prevalence of all NDDs is difficult to estimate^6^, these disorders represent a substantial global health burden, affecting approximately 15% of children and adolescents by some estimates^7^, and 1 in 345 individuals in the case of SZ^8^. The etiologies of these diverse disorders remain elusive and are thought to be multifactorial, involving both genetic and environmental risk factors. Despite epidemiological and experimental evidence implicating maternal IAV infection as a risk factor for altered offspring neurodevelopment^9–13^, the mechanisms that drive neurological dysfunction in the developing brain remain incompletely understood. Notably, data from clinical NDD populations and preclinical maternal immune activation (MIA) studies indicate that oxidative stress in the offspring brain is one of the central mechanisms contributing to neuropathogenesis^14–21^.

Oxidative stress reflects an imbalance between reactive oxygen species (ROS) production and the ability of cellular antioxidant and repair mechanisms to buffer or resolve oxidative damage, thereby contributing to cellular dysfunction, inflammation, and cell death^22–25^. Importantly, ROS are not merely downstream byproducts of inflammation. While tightly regulated ROS production supports physiological redox signaling during development^26^, excessive ROS can activate redox-sensitive inflammatory pathways, including NF-κB/MAPK and inflammasome signaling^27–29^, thereby amplifying inflammation and reinforcing a ROS-inflammation loop. Such conditions may be particularly harmful during fetal brain development, where oxidative and inflammatory insults can impair the survival of vulnerable neural populations and trigger apoptotic death, with potential long-term consequences for neurodevelopment^30–33^.

While several proinflammatory molecules have been linked to NDD-relevant mechanisms^34–36^, the molecular signals capable of driving these outcomes may extend beyond classical cytokines to include blood-derived factors that remain less well characterized. Notably, fibrinogen, a soluble plasma protein synthesized by the liver^37^, has gained increasing recognition as a potential contributor to oxidative burst-related processes, particularly through its cleavage product, fibrin^38–40^. Beyond its canonical role in hemostasis, fibrinogen has emerged as a mediator of neuroinflammatory and oxidative responses in the central nervous system, particularly when it gains access to the brain parenchyma following blood-brain barrier (BBB) disruption under pathological conditions^37,41–44^.

Once fibrinogen is cleaved into insoluble fibrin, it can bind to the CD11b/CD18 (Mac-1) integrin receptor, which is expressed on myeloid-derived cells like microglia^44,45^. This interaction may trigger redox-related and inflammatory pathways, including NADPH oxidase 2 (NOX2) activation, a key enzymatic source of ROS in microglia^46^, thereby amplifying oxidative stress and innate immune-mediated neurotoxicity^44,47–50^. Despite extensive research on the role of this blood-derived glycoprotein in oxidative stress in human and mouse models of adult neuroinflammation^38,39,51^, its potential contribution to embryonic brain development under conditions of IAV-induced MIA remains insufficiently understood. Building on our prior finding that maternal IAV exposure increases fibrinogen transfer into neurodevelopmentally relevant fetal brain regions^9^, here we examined whether fibrinogen and its insoluble derivative, fibrin, may be associated with redox-related microglial responses and apoptosis. Accordingly, we hypothesize that fibrinogen/fibrin may represent one of several blood-derived redox-modulating signals that could contribute to cellular injury pathways in the developing brain following maternal IAV-induced systemic inflammation.

## 2. Materials and methods

### 2.1. Animals

Nulliparous 9 to 10-week-old female C57BL/6JnTac mice from Taconic Biosciences (Germantown, NY) were acclimated for a minimum of one week at the University of Illinois Urbana-Champaign mouse vivarium before breeding. Females were trio-bred for three days. The presence of a vaginal plug was considered gestational day (GD) 0.5. Female body weights were recorded daily until sacrifice. All mice were maintained under a twelve-hour light/dark cycle (7:00 am lights on/ 7:00 pm lights off) and fed *ad libitum*. A total of 16 pregnant mice across two treatment groups were used to examine maternal and fetal tissues at GD/embryonic (E) day 16.5. All experimental procedures were approved by the Institutional Animal Care and Use Committee (IACUC) at the University of Illinois Urbana-Champaign.

### 2.2. Influenza A virus inoculation

IAV infection was performed as previously described^9,10^. Briefly, pregnant C57BL/6NTac mice were randomly assigned to treatment groups and inoculated on GD9.5. Under light isoflurane anesthesia, dams received an intranasal dose of 10^4^ TCID_50_ of H3N2 IAV strain X31 or sterile phosphate-buffered saline (control). Maternal and fetal tissues were collected at 7 days post-inoculation (dpi).

### 2.3. Tissue collection

Pregnant mice were euthanized by CO_2_ inhalation at 7 dpi as previously described^9^, and all tissues were collected under sterile conditions. Following excision, the uterus was transferred to a sterile petri dish containing 1X PBS (pH 7.4), where each conceptus was examined individually. Placental weight, fetal weight, crown-rump length, litter size, fetal viability, and resorptions were recorded. Fetal heads were fixed in 4% paraformaldehyde (PFA) for histology. Maternal lungs, maternal livers, and fetal livers were snap-frozen for qPCR, and fetal tails were collected and snap-frozen for sex determination.

### 2.4. Fluorescent immunohistochemistry

Fetal heads were fixed in 4% PFA (pH 7.4) for 24 h at 4°C, washed three times in PBS (pH 7.4), and cryoprotected in 30% sucrose with sodium azide at 4°C until sinking. After fixation and cryopreservation, fetal brains were dissected, embedded in OCT (Thermo Fisher Scientific, NC9159334), and stored at −80°C. Coronal sections (25 μm) were cryosectioned and cryopreserved at −20°C until immunohistochemistry (IHC). All staining configurations were performed using free-floating sections prior to slide mounting. For fibrinogen, Iba1, and phospho p47^phox^ (p47^phox^) immunofluorescence in fetal brain sections, samples were washed three times in PBS with 0.1% Triton X (0.1% PBST; Thermo Fisher Scientific, Catalog number: 34NL0800) for 5 min each time and subsequently incubated with blocking buffer (5% bovine serum albumin [Thermo Fisher Scientific, Catalog number: 126609100GM], 0.3% Triton-X 100 [Thermo Fisher Scientific, Catalog number: ICN19485450] in 1x PBS) for 1 h at room temperature. Fetal brain sections were then incubated overnight at 4°C with primary antibodies diluted in antibody buffer (1% bovine serum albumin [Thermo Fisher Scientific, Catalog number: 126609100GM], 0.1% Triton-X 100 [Thermo Fisher Scientific, Catalog number: ICN19485450] in 1x PBS). Primary antibodies: sheep anti-fibrinogen (1:500; US biological F4203–02F, coagulation factor I), rabbit anti-Iba1 (1:1000; Wako Chemicals U.S.A, Richmond, VA, Catalog number: 019–19741), goat anti-Iba1 (1:1000; Wako Chemicals U.S.A, Richmond, VA, Catalog number: 011–27991), and rabbit anti-phospho p47^phox^ (1:100; Millipore Sigma, Catalog number: SAB4504721) were used. Sections were washed three times in PBS-T and incubated with secondary antibodies, Donkey Anti-Goat AF A488 (1:1000; Jackson Immuno Research, Catalog number: 705-545-003), Donkey Anti-Rabbit AF 647 (1:500; Jackson Immuno Research, Catalog number: 711-605-152), Donkey Anti-Rabbit A488 F(ab’)_2_ fragment (1:1000; Jackson Immuno Research, Catalog number: 711-546-152), and Donkey Anti-Sheep AF A647 (1:500; Jackson Immuno Research, Catalog number: 713-605-003) for 2 h followed by nuclear staining in Hoechst (Thermo Fisher Scientific, Catalog number: H3570) for 1 min. Sections were mounted with Fluoromount-G Mounting Medium (Thermo Fisher Scientific, Catalog number: 5018788) and stored long-term at 4°C. For SOX2, NeuN, and ApopTag Red immunofluorescence, samples were washed three times in 0.1% PBST for 5 min each and permeabilized for 15 min in 0.3% Triton X-100. A brief 5-min incubation in pre-cooled ethanol:glacial acetic acid (2:1, −20°C) was followed by two additional washes in 0.1% PBST. Sections were then blocked for 1 h in 5% BSA with 0.1% Triton X-100 in PBS and then incubated overnight at 4°C with primary antibodies diluted in 1% BSA with 0.1% Triton X-100 in PBS. Primary antibodies included goat anti-SOX2 (1:200; R&D Systems, Catalog number: AF2018-SP) and chicken anti-NeuN (1:1000; GeneTex, Catalog number: GTX00837). The following day, sections were washed in 0.1% PBST and incubated for 2 h at room temperature with donkey Alexa Fluor 647 anti-goat IgG (1:1000; Jackson Immuno Research, Catalog number: 705-605-003) and donkey Alexa Fluor 488 anti-chicken IgY (1:1000; Jackson Immuno Research, Catalog number:703-545-155). After additional PBS-T washes, apoptosis labeling was performed using the ApopTag Red In Situ Apoptosis Detection Kit (MilliporeSigma S7165) according to manufacturer instructions. Briefly, sections were equilibrated in Equilibration Buffer for 10 min, incubated for 1 h at 37°C with Working Strength TdT enzyme, transferred to Stop/Wash Buffer, and washed in PBS-T before a 30 min incubation with the anti-digoxigenin conjugate. Nuclei were counterstained with Hoechst, washed, and mounted onto glass slides. Samples were cover slipped with Fluoromount-G and stored at 4°C protected from light. See section 2.12 for slide imaging.

### 2.5. BV-2 cell culture

BV-2 microglial cells (provided by Dr. Robert H. McCusker, University of Illinois Urbana-Champaign) were maintained in DMEM (SCS Cell Media Facility, UIUC) under standard humidified conditions (37°C, 5% CO_2_). For passaging or downstream assays, cells were detached using 0.25% trypsin (Gibco, Catalog number: 25300054; 5 mL, 3 min at 37°C; extended by 1 min if required for complete detachment), collected, centrifuged at 300 × g for 3 min, and resuspended in pre-warmed DMEM. Cell number and viability were determined by Trypan blue exclusion using a Countess chamber.

### 2.6. Neuronal progenitor cell (NPC) culture

Cryopreserved mouse cortical neural stem cells (NSCs; 1×10^6^ viable cells/vial; MilliporeSigma, catalog number: SCR029) were cultured as adherent monolayers on poly-L-ornithine (PLO; MilliporeSigma, catalog number: P3655) and laminin (MilliporeSigma, catalog number: L2020) coated plasticware following manufacturer recommendations. Briefly, T75 flasks and 24-well plates were coated overnight at room temperature with PLO (10 μg/mL in sterile endotoxin-free water). The next day, flasks or plates were rinsed with sterile water and then coated overnight with laminin (7 μg/mL in 1× DPBS; MilliporeSigma, catalog number: BSS-1005-B). Immediately before use, laminin was aspirated and surfaces were washed once with 1× PBS. NSCs were rapidly thawed at 37°C and transferred to a 15 mL conical tube. Cells were diluted dropwise with 9 mL of pre-warmed Neural Stem Cell Expansion Medium (MilliporeSigma, catalog number: SCM003), gently mixed without vortexing, pelleted at 300 × g for 3 min, and resuspended in 10 mL of expansion medium freshly supplemented with EGF (20 ng/mL; MilliporeSigma, catalog number: GF001) and bFGF/FGF-2 (20 ng/mL; MilliporeSigma, catalog number: GF003). Cells were plated onto coated T75 flasks and maintained at 37°C with 5% CO_2_. Medium was replaced the following day and every other day thereafter with freshly supplemented expansion medium. At ~ 90% confluence, cells were dissociated with Accutase (MilliporeSigma, catalog number: A6964; ~5 mL per T75 flask) for ~ 3 min at 37°C, or until detached. Cells were then pelleted at 300 × g for 3 min, resuspended in fresh supplemented expansion medium, and counted by Trypan blue exclusion (MilliporeSigma, catalog number: T8154). Cells were subsequently re-plated into newly coated T75 flasks for maintenance or seeded onto coated 24-well plates for downstream assays, with the same number of cells plated per well across all experimental conditions.

### 2.7. Fibrin plate preparation

This method was adapted with minor modifications from a previously published protocol^52^. To prepare fibrin-coated 96-well or 24-well plates, thrombin (2 U/mL; Sigma, Catalog number: 605157) and CaCl_2_ (14 mM; Thermo Fisher Scientific, Catalog number: L13191.0B) were first mixed in 20 mM HEPES buffer (pH 7.4; Thermo Fisher Scientific, Catalog number: NC0470071). Fibrinogen was prepared separately in cold 20 mM HEPES buffer at a working concentration of 1.08 mg/mL. Thrombin/CaCl_2_ and fibrinogen solutions were then added sequentially to each well at a 1:1 ratio. Plates were incubated for 1.5 h at 37°C, after which the coating solution was carefully aspirated. Wells were air-dried, washed once with 1× PBS, and BV-2 cells were subsequently seeded.

### 2.8. Fibrin stimulation of BV2 cells and detection of oxidant production by DHE fluorescence

This method was adapted with minor modifications from^53^. For oxidant detection, BV-2 cell suspensions were incubated with 5 μM of dihydroethidium (DHE, Thermo Fisher Scientific, Catalog number: D23107) and incubated for 30 min at 37°C protected from light. DHE-loaded cells were then plated at 100,000 cells/well (100 μL/well) in black, clear-bottom 96-well plates (Thermo Fisher Scientific, Catalog number: 07-000-630) pre-coated with or without fibrin and incubated for 24 h at 37°C. After incubation, medium was carefully aspirated and cells were fixed with 100 μL/well of 4% formaldehyde (Thermo Fisher Scientific, Catalog number: 28906) for 10 min at room temperature protected from light. Wells were then washed twice with 1 x PBS, and 100 μL PBS was left in each well for immediate quantification with plate reader (Multi-Mode Microplate Reader, Agilent BioTek Synergy H1, Serial #: 23121806) at 518/606 nm (Ex/Em).

### 2.9. Treatment of NPCs with BV2-conditioned media

After 48 h in culture, NPCs cultured on μ-Plate 24 Well Black ibiTreat plates with #1.5 polymer coverslips (ibidi, Catalog number: 82426) were exposed to BV-2-conditioned medium. The existing NPC growth medium was aspirated, and cells were gently rinsed once with sterile 1× PBS. Cultures were then incubated in a medium consisting of 50% BV-2-conditioned medium and 50% fresh supplemented NPC growth medium. Conditioned media collected from either fibrin-treated or control BV-2 cultures was applied as indicated. After 30 min of exposure, cells were fixed and processed for CC3/SOX2/Hoechst staining.

### 2.10. In vitro apoptosis detection and immunocytochemical characterization of NPCs

Following exposure to BV-2-conditioned media, NPCs were fixed in 4% formaldehyde for 10 min at room temperature. Following fixation, cells were rinsed twice in 1× PBS, permeabilized in 0.3% Triton X-100 in PBS for 15 min at room temperature, and rinsed again twice in 1× PBS. Cells were then incubated for 5 min in pre-cooled ethanol:glacial acetic acid (2:1) at − 20°C, drained without allowing the wells to dry, and rinsed twice in 1× PBS. Cells where then incubated for 1 h in 5% BSA with 0.1% Triton X-100 in PBS, and washed twice before overnight incubation at 4°C with primary antibodies diluted in 1% BSA with 0.1% Triton X-100 in PBS. Primary antibodies included goat anti-SOX2 (1:200; R&D Systems, Catalog number: AF2018-SP), and Cleaved Caspase-3-Asp175 (1:400; Cell Signaling, Catalog number: 9661S). The following day, cells were washed in 0.1% PBS-T and incubated for 1 h at room temperature with donkey Alexa Fluor 647 anti-goat IgG (1:1000), and donkey Alexa Fluor 594 anti-rabbit IgG (1:1000, Jackson Immuno Research, Catalog number: 711-585-152). After three final washes in 1× PBS, samples were counterstained with Hoechst and imaged on a Leica THUNDER imaging system (see section 2.12).

### 2.11. Proteome Profiler cytokine array analysis of BV-2 cell culture supernatants

Supernatants collected from BV-2 cells cultured on fibrin-coated plates or uncoated control wells, as described above, were analyzed using the Proteome Profiler Mouse Cytokine Array Panel A kit (R&D Systems, Catalog no. ARY006). The assay was performed according to the manufacturer’s protocol with minor adjustments. Briefly, 2 mL of blocking solution was added to each well of the 4-well multi-dish, and each membrane was placed individually in a well with the identification number facing upward. Membranes were incubated for 1 hour on a rocking platform shaker. During membrane blocking, sample mixtures were prepared by combining 1 mL of pooled cell culture supernatant from each experimental condition with 0.5 mL of the provided assay solution. The reconstituted Detection Antibody Cocktail was then added to each sample mixture, mixed, and incubated for 1 hour at room temperature. After blocking, the blocking solution was removed and replaced with the corresponding sample/antibody mixture. Membranes were incubated overnight at 4°C on a rocking platform shaker. The following day, membranes were transferred to individual containers and washed three times with 1X wash solution for 10 mins each on a rocking platform shaker. Streptavidin-HRP was then diluted according to manufacturer’s instructions and added to each membrane. Membranes were incubated with Strep-HRP for 30 mins at room temperature on a rocking platform shaker, followed by three additional washes. For chemiluminescent detection, each membrane was placed on the bottom sheet of a plastic sheet protector with the identification number facing upward and covered with 1 mL of freshly prepared Chemi Reagent Mix. After a 1-min incubation, excess reagent was removed, and membranes were imaged for 3 mins using an iBright1500 imaging system (Invitrogen). Pixel density for each cytokine target was quantified using ZEISS ZEN 3.0 Blue software to determine approximate abundance, expressed as arbitrary units (AU). Because the proteome profiler array was used as a targeted exploratory screen and few analytes showed large fibrin-induced changes, raw signal intensities were secondarily evaluated using a descriptive threshold of ≥ 15% change relative to non-fibrin-stimulated BV-2 controls. Analytes meeting this threshold were considered fibrin-responsive candidates.

### 2.12. Imaging & Image Analysis

All immunofluorescent images were acquired using a Leica THUNDER Imager equipped with LED illumination (emission wavelengths: 400, 450, 550, and 635 nm), long-pass filter cubes (440, 510, 590, and 700 nm), and a Leica K5 monochrome sCMOS camera. Images were captured using a 20x/0.40 NA HC PL FL air objective with adjustable correction collar (WD 6.2–7.5 mm). .Mean fluorescence intensity (MFI) of immunolabeling was quantified in Fiji (ImageJ version 1.54p) and expressed as MFI/mm^2^. SOX2 colocalized with ApopTag Red, NeuN colocalized with ApopTag Red, Iba1 colocalized with fibrinogen, and Iba1 colocalized with p47^phox^ were manually counted and normalized by area (cells/mm^2^).

Automated quantification of SOX2^+^ and NeuN^+^ populations within the subventricular zone (SVZ) and across the hemisphere was performed using Cellpose (www.cellpose.org; version 4, CP-SAM model)^54,55^ with outputs similarly normalized by area. For image preprocessing, raw fluorescence images were processed in Fiji using the following sequential workflow: (1) contrast-limited adaptive histogram equalization (CLAHE; block size = 127 pixels, 256 histogram bins)) to enhance local contrast while minimizing noise amplification; (2) unsharp mask filtering (radius = 2.0 pixels, mask weight = 0.6) to sharpen cellular boundaries. Preprocessed images were segmented using the CP-SAM pretrained model with the following parameters: cell diameter = 61 pixels, flow threshold = 0.7, cellprob threshold = 0.4, optimized by manual validation across representative images. Segmentation masks were imported into Fiji using the BIOP Label Image to ROIs plugin, and cell counts were normalized by the measured area of each region of interest (cells/mm^2^).

For *in vitro* NPC culture experiments, nuclear segmentation was performed using Cellpose version 3.1.1 (cyto3 model) with GPU acceleration. Hoechst images were preprocessed using background subtraction (Gaussian approximation, sigma = 50 pixels) followed by CLAHE (block size = 127 pixels, 256 histogram bins, maximum slope = 3.5). Segmentation parameters were set as follows: cell diameter = 35.8 pixels, flow threshold = 0.5, cellprob threshold = 0, optimized by manual validation across representative images. Colocalization of cleaved caspase 3 and SOX2 was quantified by measuring mean fluorescence intensity of raw marker channels within each Cellpose-defined nuclear mask. Fixed intensity thresholds (cleaved caspase 3 = 50, SOX2 = 80; 16-bit intensity units, approximately 3–4 times median background) were applied uniformly across all samples, and results were normalized to positive cells per 100,000 nuclei to account for variable cell density across wells. All MFI measurements, manual cell counts, and automated analyses were performed under fully blinded conditions. Fetal brain regions of interest (ROIs) were identified using the Allen Mouse Brain developmental reference atlas^56^ and^57^.

### 2.13. Sex determination

SRY genotyping was performed to determine fetal sex using a rapid alkaline tail lysis protocol adapted from^58^, followed by multiplex PCR targeting the Y chromosome specific *Sry* gene and the autosomal control gene *Myog*. Tail tips (1–2 mm) were digested in 50 mM NaOH at 95°C for 10 minutes, neutralized with 1 M Tris (pH 8), and centrifuged. The resulting supernatant served as the DNA template. PCR reactions (20 μL) contained Hot Start Taq Master Mix (VWR, Catalog number: 97068–148) and four primers. **Supplemental Table S1** contains the list of primers used for gene expression, acquired from Integrated DNA Technologies (IDT) Custom DNA Oligos. Thermal cycling consisted of 95°C for 5 minutes; 95°C for 30 seconds, 35 cycles of 65°C for 30 seconds, and 72°C for 45 seconds; and a final extension at 72°C for 10 minutes. Amplicons were resolved on a 2% agarose gel containing SYBR Safe (Invitrogen, Catalog number: S33102) and visualized under fluorescence. The *Myog* product (~ 250 bp) served as an internal control, while the presence of the *Sry* amplicon (~ 450 bp) identified male (XY chromosomes) samples. Samples displaying only the *Myog* band were classified as female (XX chromosomes; **Supp. Fig. S1F**).

### 2.14. Quantitative real-time PCR

RNA from fetal and maternal livers was isolated using TRIzol via the TRI reagent protocol from Invitrogen (Carlsbad, CA). cDNA was produced using the High-Capacity cDNA reverse transcription kit (Applied Biosystems) following the manufacturer’s instructions, and qPCR was performed on QuantStudio 5 using SYBR Green Master Mix (Applied Biosystems). The 2-ΔΔCt method was used to analyze samples relative to *Hprt1* housekeeping gene.

### 2.15. Statistics

Unless otherwise specified, data were analyzed using GraphPad Prism 9 software (San Diego, CA) with significance set at α = 0.05. Con and IAV treatment groups were compared for maternal body weight trajectories using a repeated-measures two-way ANOVA with Šídák’s post hoc correction for multiple comparisons. For litter size, viability, and resorption, group differences were evaluated using the Mann-Whitney U test. Comparisons between two independent conditions, including maternal qPCR analyses, *in vitro* experiments, and p47^phox^ analyses, were analyzed using an unpaired, two-tailed t test after confirming homogeneity of variance. Homogeneity of variance was assessed using an F test, and Welch’s correction was applied when variances differed between groups.

For fetal and placental weight/size measurements, all fetuses and placentas in each litter were included and data were analyzed using a mixed-effects model with litter as a random effect. For all other fetal data, sex and treatment effects were assessed using a linear mixed-effects modeling framework to quantify the independent and interacting contributions of both factors while accounting for the non-independence of pups originating from the same dam. A minimum of one randomly selected male fetus and one randomly selected female fetus from each litter were included. Male and female fetal measurements were reorganized into long format and analyzed jointly to preserve statistical power and enable direct testing of sex-by-treatment interactions. Treatment and sex were modeled as fixed effects, including their interaction term, and dam/litter was modeled as a random intercept to account for litter-level clustering. Models were fit using restricted maximum likelihood (REML) with the bobyqa optimizer, and Type III ANOVA with Satterthwaite’s approximation was used to evaluate the significance of main effects and interactions. When a significant effect was detected, post hoc comparisons were performed using estimated marginal means, including Tukey-adjusted contrasts among all treatment-sex combinations and Holm-adjusted pairwise tests examining treatment effects within each sex, and sex effects within each treatment. All analyses were conducted in R using the packages lme4, lmerTest, emmeans, dplyr, and tidyr.

Each statistical test is indicated in the figure legend. Data are expressed as means ± SEM, unless otherwise stated.

## 3. Results

### 3.1. Gestational IAV infection recapitulates clinical phenotypes and induces fetoplacental growth restriction

To confirm that our gestational H3N2 X31 infection paradigm reliably induced a maternal disease state, and to place downstream fetal brain analyses within a validated MIA context, we first assessed maternal physiological readouts together with fetoplacental growth metrics following inoculation. Relative to controls, IAV dams exhibited reduced gestational body weight gain after inoculation (**Supp. Fig. S1A**). In parallel, lung viral qPCR confirmed the presence of viral nucleoprotein RNA in X31-infected dams (**Supp. Fig. S1B**), supporting successful maternal infection. These findings are consistent with our previous characterizations of this gestational flu infection model, in which attenuated maternal weight gain coincides with pulmonary histopathology, splenomegaly, and systemic inflammatory cytokine responses^9,10^.

We next examined whether maternal illness was accompanied by evidence of fetoplacental growth impairment. Consistent with prior work showing that gestational IAV can impose a growth restrictive intrauterine environment^9,59–61^, maternal flu was associated with reduced fetal weight, shorter crown-to-rump length, and decreased placental weight (**Supp. Fig. S1C-E**), while gross litter outcomes (e.g., number and viability of pups per litter) were largely preserved (**Supp. Table S2**). These findings replicate our previous report of intrauterine growth restriction^9^, a systemic consequence of gestational infection that may reshape the developmental environment experienced by the fetus^15,62,63^.

Given these effects, we next asked whether gestational IAV infection engaged a measurable acute phase inflammatory response, and therefore quantified acute phase protein gene expression in the maternal and fetal liver, including fibrinogen related transcripts. These proteins are canonical hepatic markers of systemic inflammation, and their production is commonly upregulated in response to inflammatory challenge, particularly through IL-6-dependent signaling pathways^64,65^. At 7 dpi, the maternal liver showed a treatment-dependent increase in *Hp* (haptoglobin) expression and a concomitant reduction in *Fga* (fibrinogen alpha chain), while other acute-phase transcripts (*Fgb, Fgg, and Crp*) were not detectably altered (**Supp. Fig. S2A-E**). This selective acute phase signature is consistent with time dependent regulation of hepatic inflammatory programs^66^, reflecting an ongoing or partially resolving systemic acute-phase response at the time of collection (7 dpi)^66^. In contrast, the fetal liver did not exhibit treatment or sex-dependent differences in acute-phase gene expression (**Supp. Fig. S2F-J**), indicating that this transcriptional response was largely restricted to the maternal compartment.

Collectively, these data show that gestational IAV infection recapitulated the expected maternal disease phenotype and associated fetoplacental growth restriction^9,10^, consistent with clinically relevant systemic inflammation following respiratory viral infection.

### 3.2. Maternal IAV infection increases fibrinogen accumulation and colocalization with Iba1^+^ cells across key regions of the developing fetal brain

**Figure 1:**
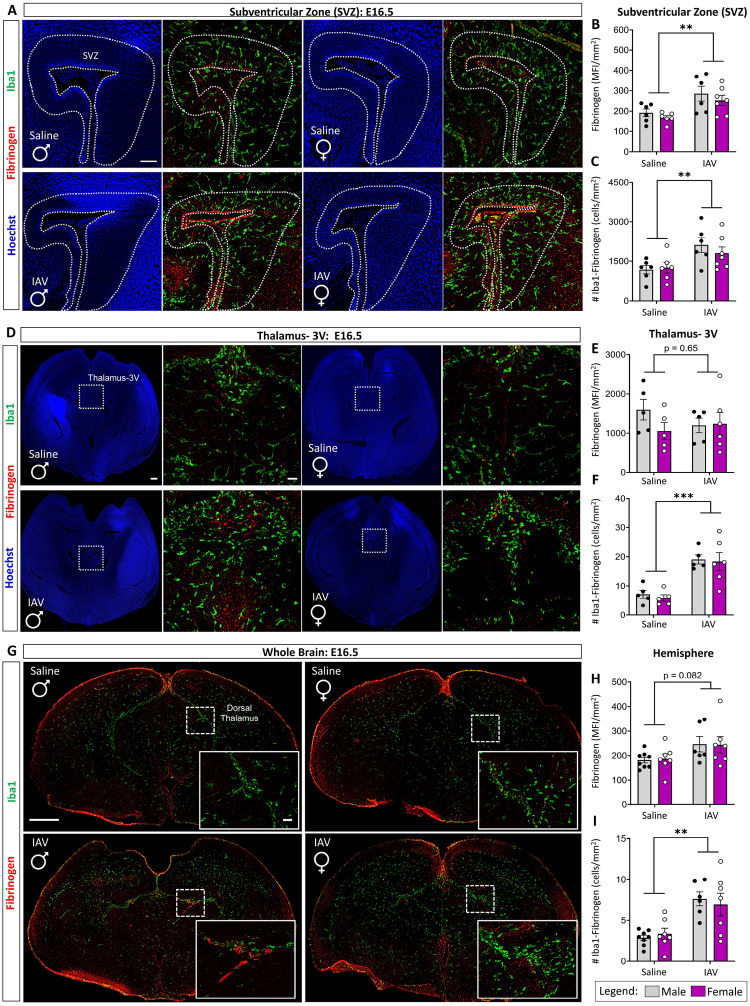
Maternal IAV infection induced region-specific increases in fibrinogen accumulation and fibrinogen-associated Iba1^+^ cells in the developing fetal brain. (**A**) Representative high-magnification image of the SVZ (outlined in Hoechst image) illustrating fibrinogen (red) and Iba1 (green) spatial associations across sex (male vs female) and treatment group (Saline vs IAV). (**B**) Fibrinogen MFI/mm^2^ in the SVZ was elevated in IAV fetuses regardless of sex. (**C**) Number of Iba1^+^cells associated with fibrinogen in the SVZ also increased in IAV fetuses compared to Saline, with no significant sex effect. (**D**) Representative images of the Thalamus-3V region, outlined in the Hoechst image on the right, and magnified on the left showing fibrinogen (red) and Iba1 (green). (**E**) Quantification of fibrinogen MFI/mm^2^ in the Thalamus-3V indicates no treatment or sex differences. (**F**) In contrast, number of Iba1^+^ cells associated with fibrinogen in the Thalamus-3V increased in IAV fetuses compared to Saline, with no significant sex effect. (**G**) Representative coronal fetal brain sections, with the dorsal thalamus outlined; insets highlight regions where fibrinogen is spatially associated with Iba1^+^ cells. (**H**) Quantification of fibrinogen MFI/mm^2^ in the whole hemisphere showed no differences across treatment or sex, although IAV fetuses tended to have more fibrinogen fluorescence compared to Saline (p = 0.08). (**I**) Number of Iba1^+^ cells spatially associated with fibrinogen in the whole hemisphere increased in IAV fetuses compared to Saline, with no significant sex effect, similar to the thalamus and SVZ. Data were analyzed using linear mixed-effects models with treatment (saline vs IAV) and sex (male vs female) as fixed factors and dam as a random effect to account for litter effects. Type III ANOVA with Satterthwaite approximation tested fixed effects, and post-hoc contrasts were obtained using estimated marginal means with Holm/Tukey correction. Data shown as mean ± SEM. Circles represent individual fetuses; n/sex/group: Saline: n = 5–8, IAV: n = 5–7. * = p < 0.05, ** = p < 0.01. Scale bars: (**A**) 50 μm; (**D**) whole hemisphere = 200 μm, inset = 50 μm; (**G**) whole hemisphere = 500 μm, inset = 50 μm. SVZ = Subventricular Zone; Thalamus-3V =Thalamus-third ventricle.

Growing evidence supports fibrinogen as a central mediator of neuroinflammatory pathology in neurodegenerative conditions or injury^67–69^. When brain vascular integrity becomes compromised in these conditions, fibrinogen may gain access to the brain parenchyma and, upon conversion to fibrin, engage microglia, promoting inflammatory signaling and ROS production that can exacerbate neural injury^38,43,53^. The extent to which this process occurs during insults in *early* life is unclear. In line with our previous work showing that IAV-induced MIA increases fibrinogen leakage into key fetal neurodevelopmental regions^9^, here we again observed enhanced fibrinogen accumulation in the SVZ in fetuses from IAV-exposed dams (Fig. 1A–B). This feature was accompanied by a greater colocalization of fibrinogen with Iba1^+^ microglial cells (Fig. 1C). Based on this pattern, we next asked whether fibrinogen accumulation and its relationship with microglia could be extended to other neurodevelopmentally relevant brain regions.

We extended our analysis to the thalamus-third ventricle (Thalamus-3V) interface (Fig. 1D). During late gestation in mice (~ E17), and the late second-to-early-third trimester in humans (~ 24 weeks), the thalamus is a key hub for the establishment of thalamocortical circuits that support sensory integration and higher-order cognitive processing^70–74^. In parallel, the third ventricle serves as a critical CSF-brain interface regulating regional neurodevelopment and barrier-associated signaling^75–77^. Quantification of fibrinogen MFI/mm^2^ within the fetal Thalamus-3V revealed no treatment or sex effects (Fig. 1E). Still, fibrinogen colocalization with Iba1^+^ microglia was greater in the IAV group compared to controls (Fig. 1F). We then examined whether a similar pattern was evident at a broader anatomical scale across the whole hemisphere (Fig. 1G). Consistent with the Thalamus-3V analyses above, quantification of fibrinogen MFI/mm^2^ across the hemisphere did not reveal a significant effect of treatment (Fig. 1H). Nevertheless, the number of Iba1^+^ cells associated with fibrinogen was increased in IAV fetuses relative to controls (Fig. 1I), indicating that enhanced fibrinogen-microglial cell association is broadly detectable across the E16.5 brain. Because this hemisphere measurement includes regions that showed significant effects in targeted analyses, the observed increase may reflect regionally localized fibrinogen-microglia interactions rather than a uniform hemisphere-wide change. Taken together, these data indicate that maternal IAV infection elicits site-specific fibrinogen accumulation in the SVZ, with broad increases in fibrinogen-microglia association across multiple regions critical for neurodevelopment.

### 3.2. Maternal IAV infection increases Iba1^+^/p47 ^phox +^ cell density in selected fetal brain regions without evidence of fetal sex-dependent effects

Given that fibrinogen-microglial engagement can trigger redox signaling and amplify neuroinflammatory programs^47,69,78,79^, we next asked whether maternal flu may affect the abundance of fetal p47^phox^, a key cytosolic organizer subunit of NOX2 that supports inducible ROS generation in myeloid cells, including microglia^80–83^. Mechanistically, NOX2 activity is not solely dictated by steady-state protein abundance; it is enabled through stimulus-dependent assembly of the oxidase at the membrane. In its basal state, the membrane core (NOX2/gp91^phox^ with p22^phox^) remains largely inactive until cytosolic subunits are mobilized. Upon activation, p47^phox^ undergoes phosphorylation-dependent conformational “opening,” exposing SH3 domains that promote docking to p22^phox^ and coordinating the recruitment of additional cytosolic partners (including p67^phox^ and p40^phox^), together with the small GTPase Rac, to form an active enzymatic complex capable of generating superoxide^46,84–87^. Thus, we tested whether maternal IAV infection alters fetal brain p47^phox^ phosphorylation, a key regulatory event that promotes p47^phox^ translocation and supports stimulus-dependent assembly of the NOX2 complex, favoring inducible ROS generation^86,87^.

**Figure 2:**
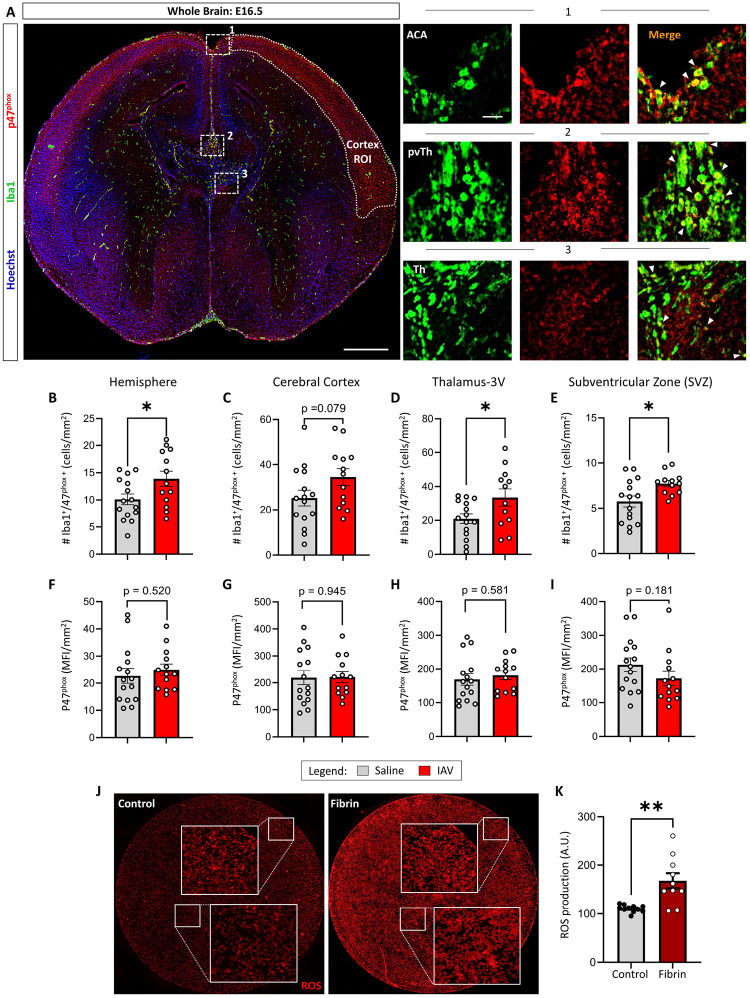
Maternal flu increases the number of Iba1^+^/p47^phox+^ cells in selected fetal brain regions, along with an increase in ROS production in BV-2 cells *in vitro*. (**A**) Representative coronal fetal brain sections showing staining patterns of Iba1 (green) and p47^phox^ (red), across brain regions: whole brain, cerebral cortex ROI, medial cortex/anterior cingulate area (ACA; dotted box 1), periventricular thalamus (pvTh; dotted box 2), and lateral thalamic parenchyma (Th; dotted box 3). Magnified images of each box (1 = ACA, 2 = pvTh, 3 = Th) across the green (Iba1) and red (p47^phox^) channels are shown to the right, with arrows indicating bright double-positive cells. (**B-E**) Counts of Iba1-p47^phox^ double-positive cells in the whole hemisphere, cerebral cortex, thalamus-3^rd^ ventricle interface, and subventricular zone. In **B**, **D**, and **E**, Iba1-p47^phox^ double-positive cells were increased in the IAV group compared with Saline, whereas counts did not reach significance (p = 0.08) in **C**. Quantification of p47^phox^ MFI/mm^2^ across brain regions (**F-I**) revealed no significant differences among treatment groups in any region examined. (**J**) Representative images of ROS production in BV-2 cells, assessed by dihydroethidium (DHE) oxidation, under control conditions vs fibrin exposure. (**K**) Quantification of DHE oxidation-derived fluorescence shows increased ROS production in BV-2 cells exposed to fibrin compared with control. All data were compared using an unpaired, two-tailed t test with Welch’s correction. In **B-I** circles represent individual male and female fetuses; n per group: Saline = 15, IAV = 12–13. In **K**, circles represent individual wells (technical replicates); n per group: Control: n = 11, Fibrin: n = 10. All data are represented as mean ± SEM. Scale bars: 500 μm and 30 μm (insets). ACA = Anterior cingulate area; Thalamus-3V = Thalamus-third ventricle.

We first quantified fetal brain Iba1^+^/p47^phox+^ cell density together with overall p47^phox^ fluorescence intensity at E16.5 across the whole hemisphere, cerebral cortex, Thalamus-3V interface, and SVZ (Fig. 2A–I). Fetuses from IAV-infected dams showed increased Iba1^+^/p47phox^+^ cell density for all regions except the cortex when males and females were analyzed together (Fig. 2B–E). In contrast, overall p47^phox^ fluorescence intensity (MFI/mm^2^) was not altered by maternal treatment in any region examined (Fig. 2F–I). To determine whether these outcomes were modified by fetal sex, we next analyzed males and females separately. This analysis did not reveal significant fetal sex effects or treatment × sex interactions for either regional p47^phox^ MFI/mm^2^ or Iba1^+^/p47^phox+^ cell density (**Supp. Fig. S3**). As p47^phox^ functions as a regulatory subunit of the NOX2 complex, this finding indicates a greater abundance of fetal Iba1^+^ cells with p47^phox^-associated pro-oxidative potential, without a generalized increase in p47^phox^ activity.

Because NOX2 activation is highly dynamic^86,88^ and depends on stimulus-dependent assembly of the oxidase complex rather than steady-state signal intensity, we next used an *in vitro* proof-of-concept approach to determine whether fibrin is sufficient to enhance oxidative responses in microglial-like cells. Specifically, we quantified ROS generation in BV-2 microglial-like cells using DHE oxidation as a readout (Fig. 2J). Under these conditions, exposure to fibrin increased oxidative DHE-derived fluorescence relative to control (Fig. 2K).

Together, these findings suggest that maternal flu does not induce broad changes in fetal brain p47^phox^ activity at E16.5, but is associated with increases in the density of pro-oxidative microglia/macrophages (i.e., Iba1^+^ cells colocalizing with p47^phox^) in the same brain regions that demonstrate increased Iba1-fibrinogen colocalization. In parallel, the fibrin-induced increase in DHE oxidation in BV-2 microglial-like cells provides proof-of-concept evidence that fibrin-associated cues can enhance oxidative responses in microglial-like cells, supporting the plausibility of a relationship between fibrin exposure and redox-related microglial responses.

### 3.3. SOX2^+^ neuronal progenitor density shows region- and sex-specific variation at E16.5

Given that MIA can elevate inflammatory and redox stress pathways^21,89–91^, and that oxidative stress has been implicated in regulating neural progenitor dynamics^92–95^, we next asked whether maternal flu might affect fetal neural progenitor abundance. To assess this, we quantified SOX2, a transcription factor widely used to identify neural progenitor and stem-like populations in the developing brain^96,97^.

**Figure 3:**
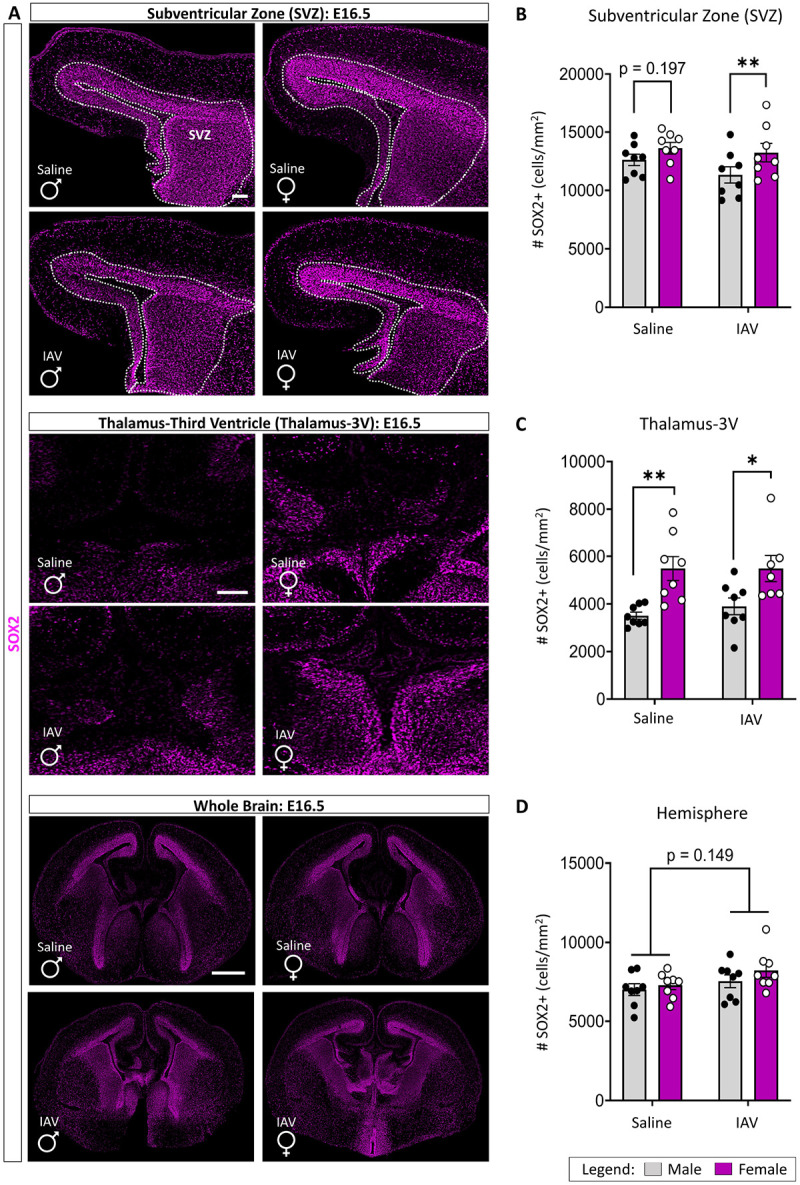
Region- and sex-specific patterns of SOX2^+^ progenitor density in fetal brains. (**A**) Representative coronal sections of the SVZ (upper panel), thalamus-3V interface (middle panel), and whole brain (lower panel) showing SOX2^+^ neural progenitors (purple) across sex and treatment groups. (**B**) Within the SVZ, SOX2^+^ cell density showed a main effect of sex, with the male-female difference most evident within the IAV group. Males from IAV-infected dams showed lower SOX2^+^ density than females from the same group, whereas saline males and females did not differ. Neither main effect of treatment nor treatment-by-sex interaction reached significance. (**C**) Within the Thalamus-3V, females exhibited higher SOX2^+^ cell density than males in both Saline and IAV groups, reflecting a robust main effect of sex. Neither main effect of treatment nor treatment-by-sex interaction reached significance. (**D**) Across hemispheres, no main effect reached significance. SOX2^+^ cells were counted using CellPose and analyzed using linear mixed effects models with treatment (saline vs IAV) and sex (male vs female) as fixed factors and dam as a random effect to account for litter effects. Type III ANOVA with Satterthwaite approximation tested fixed effects. Data shown as mean ± SEM. Circles represent individual fetuses; n/sex/group = 8. Scale bars: SVZ = 300 μm, Thalamus-3V = 200 μm, Whole Brain = 500 μm. SVZ = Subventricular Zone; Thalamus-3V = Thalamus-third ventricle.

E16.5 SOX2^+^ progenitor cell density was quantified in the SVZ, Thalamus-3V interface, and whole hemisphere, with sex included as a biological factor (representative images in Fig. 3A). In the SVZ, there was a significant main effect of sex, driven primarily by lower SOX2^+^ density in IAV males compared with IAV females; no significant sex difference was detected in saline controls (Fig. 3A **upper panel &** 3B). This effect occurred in the absence of a significant main effect of treatment or treatment x sex interaction. At the Thalamus-3V interface, shifts in SOX2^+^ cell density were also sex-dependent, with females displaying higher density than males in both saline and IAV conditions (Fig. 3A **middle panel &** 3C), suggesting a regional sex effect that was not restricted to maternal flu. In contrast, SOX2^+^ cell density measured across the embryonic hemisphere did not show a detectable effect of treatment nor sex (Fig. 3A **lower panel &** 3D).

Taken together, these findings indicate that fetal SOX2^+^ progenitor density at E16.5 is not uniformly affected by maternal flu. Instead, we observe region-specific sex-related patterns, with the most pronounced male-female difference observed in the SVZ of fetuses from IAV-infected dams. These results suggest that progenitor abundance is regionally patterned and sex-dependent rather than broadly altered following maternal viral challenge.

### 3.4. Gestational IAV infection selectively increases apoptosis in male SOX2^+^ progenitors.

**Figure 4:**
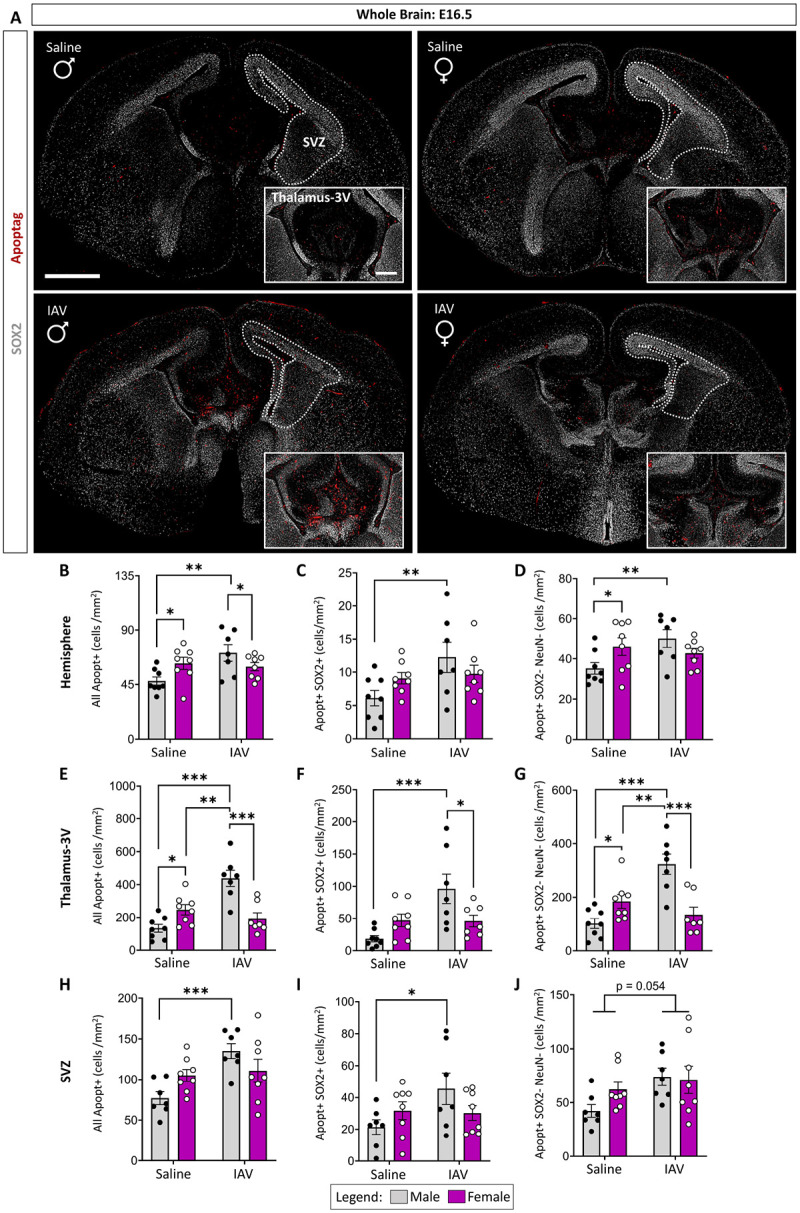
Maternal IAV infection increases DNA fragmentation in male fetal brains, with prominent involvement of SOX2^+^progenitors. (**A**) Representative coronal fetal brain sections at E16.5 showing SOX2^+^neural progenitors (gray) and ApopTag^+^ apoptotic cells (red) across sex and treatment groups. Main panels show the embryonic whole brain with the SVZ outlined (dotted line), and insets show the Thalamus-3V interface. **In the embryonic hemisphere**: (**B**) males from IAV-infected dams showed increased total apoptosis compared with males from control dams, whereas females showed no detectable treatment effect. Sex comparisons showed higher apoptosis in saline females than saline males and higher apoptosis in IAV males than IAV females. (**C**) SOX2^+^apoptotic progenitors were increased in IAV males relative to saline males, with no detectable treatment effect in females. (**D**) Apoptotic cells excluding SOX2 and NeuN populations were also increased in IAV males relative to saline males, whereas females showed no detectable treatment effect. Sex comparisons showed higher apoptosis in saline females than saline males. **In the Thalamus-3V interface**: (**E**) total apoptosis was increased in IAV males relative to saline males, with no detectable treatment effect in females. Sex comparisons showed higher apoptosis in saline females than saline males and higher apoptosis in IAV males than IAV females. (**F**) SOX2^+^ apoptotic progenitors were increased in IAV males relative to saline males, with no detectable treatment effect in females. Sex comparisons showed higher apoptosis in IAV males than IAV females. (**G**) Apoptotic cells excluding SOX2 and NeuN populations were similarly increased in IAV males relative to saline males, with no detectable treatment effect in females. Sex comparisons showed higher apoptosis in saline females than saline males and higher apoptosis in IAV males than IAV females. **In the SVZ**: (**H**) total apoptosis was increased in IAV males relative to saline males, whereas females showed no detectable treatment effect. (**I**) SOX2^+^apoptotic progenitors were increased in IAV males relative to saline males, with no detectable treatment effect in females. (**J**) Apoptotic cells excluding SOX2 and NeuN populations did not show significant effects of treatment or sex. All data were analyzed using linear mixed-effects models with treatment (saline vs IAV) and sex (male vs female) as fixed factors and dam as a random effect. Fixed effects were evaluated using Type III ANOVA with Satterthwaite approximation; post-hoc contrasts were obtained using estimated marginal means with Holm/Tukey correction. Data represent mean ± SEM; circles represent individual fetuses. n/sex/group = 7–8. * = p < 0.05, ** = p < 0.01, *** = p < 0.001. Scale bars: 500 μm and 200 μm (insets). SVZ = Subventricular Zone; Thalamus-3V =Thalamus-third ventricle.

Given that SOX2^+^ progenitor density showed regionally selective changes rather than broad alterations across the fetal brain, we next asked whether maternal flu was associated with altered fetal progenitor cell survival, a process that may be sensitive to inflammatory and redox-related stress pathways during MIA^21,89,98–100^. To test this, we combined ApopTag labeling with SOX2 immunolabeling to assess apoptosis within the SOX2^+^ progenitor population; we included NeuN to also evaluate apoptosis in postmitotic neurons. Representative coronal brain sections show overall ApopTag^+^ profiles with SOX2 and NeuN co-labeling across treatment and sex, highlighting SVZ-associated progenitor domains and insets marking the Thalamus-3V region (Fig. 4A, **Supp. Fig. S4A**).

In the whole E16.5 hemisphere, maternal flu increased apoptosis in the fetal brain in a sex-dependent manner. Total ApopTag^+^ cell density was elevated in IAV males relative to saline males, whereas females showed no treatment-dependent change (Fig. 4B). A similar IAV male-specific increase was observed among apoptotic SOX2^+^ progenitors (Fig. 4C) and ApopTag^+^ cells lacking both SOX2 and NeuN labeling (Fig. 4D). The same pattern was observed for all ApopTag^+^ cell subsets the Thalamus-3V interface (Fig. 4E–G), where differences among saline and IAV males were the most pronounced, and within-treatment differences between males and females was further accentuated.

The SVZ showed a related male-biased apoptotic pattern, but with a narrower group distribution than that observed at the Thalamus-3V interface. Again, total SVZ ApopTag^+^ cell density was increased in males from IAV dams compared with male controls, whereas females showed no detectable treatment-related difference (Fig. 4H), and apoptotic SOX2^+^ progenitors showed a similar IAV male-specific increase (Fig. 4I). In contrast, ApopTag^+^ cells lacking SOX2 and NeuN labeling did not differ in the SVZ, despite a directional trend consistent with the pattern observed in other regions (Fig. 4J).

In contrast, apoptosis among NeuN^+^ postmitotic neurons was not altered by treatment or sex in any region (**Supp. Fig. S4**), and overall number of apoptotic mature neurons was notably low compared to apoptotic progenitors.

Overall, these findings indicate that maternal flu increases apoptosis in the E16.5 fetal brain in a sex-dependent manner, with males showing greater vulnerability across multiple regions. This response was most consistently detected in SOX2^+^ progenitors, whereas NeuN^+^ postmitotic neuronal apoptosis was not altered.

### 3.5. Conditioned medium from fibrin-challenged BV-2 microglia increases apoptosis-associated readouts in NPCs in vitro

Given the increased fibrinogen-Iba1 colocalization observed in fetal brain regions after maternal flu (Fig. 1), together with increased Iba1^+^/p47^phox+^ cell density in selected regions (Fig. 2) and evidence of male-biased apoptosis among SOX2^+^ progenitors *in vivo* (Fig. 4), we next tested whether soluble factors released by fibrin-challenged BV-2 cells could influence neural progenitor cell (NPC) survival. We hypothesized that fibrinogen/fibrin exposure engages microglia-associated redox and inflammatory pathways, thereby altering the soluble microenvironment in ways that may increase apoptotic vulnerability in NPCs. As shown in Fig. 2, BV-2 cells produced more ROS in response to fibrin exposure *in vitro*.

To determine whether fibrin also altered the release of soluble inflammatory mediators, conditioned media from BV-2 cultures challenged with or without fibrin was analyzed using a targeted inflammatory protein array. Of the 40 proteins included in the array, only 8 exhibited at least a 15% change relative to non-fibrin-stimulated controls. These included G-CSF, ICAM-1, IL-3, IP-10 (CXCL10), M-CSF, TIMP-1, IL-1ra, and RANTES (CCL5; **Supp. Fig. S5**). Most of these factors showed lower relative abundance after fibrin exposure, whereas only G-CSF and ICAM-1 were increased. These proteins span multiple functional categories, including pro-inflammatory chemokines (CXCL10, CCL5), hematopoietic/myeloid-associated trophic factors (G-CSF, IL-3, M-CSF), the tissue-remodeling mediator TIMP-1, the adhesion/transmigration molecule ICAM-1, and the counter-regulatory IL-1 receptor antagonist IL-1ra. Thus, fibrin stimulation was associated with a limited and functionally heterogeneous shift in soluble inflammatory and immune-regulatory proteins.

**Figure 5:**
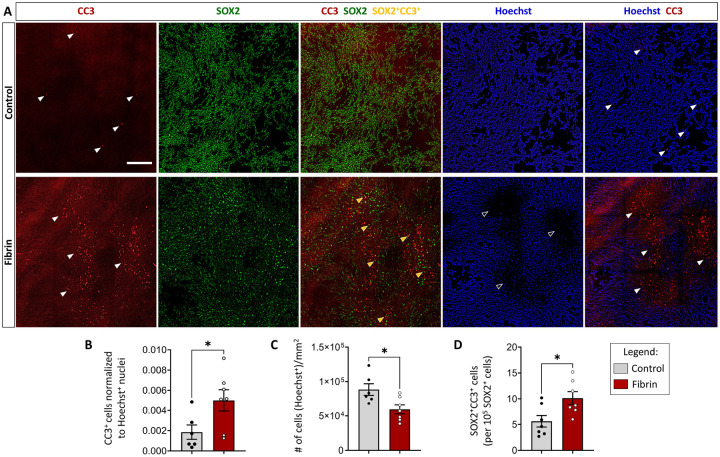
Conditioned medium from fibrin-stimulated BV-2 cells increases CC3 immunolabeling and reduces NPC cell number. (**A**) Representative ICC images of SOX2^+^ NPC cultures exposed to conditioned media collected from control BV-2 cells or fibrin-stimulated BV-2 cells. Cultures were immunostained for SOX2 to identify NPCs and CC3 to assess apoptotic marker labeling, with Hoechst used to label nuclei. (**B**) Quantification of CC3^+^ NPCs normalized to Hoechst^+^ nuclei. Exposure to conditioned medium from fibrin-stimulated BV-2 cells increased CC3^+^ NPC labeling relative to control conditioned media. (**C**) Quantification of Hoechst^+^ nuclei per mm^2^ following conditioned media exposure. NPC cultures exposed to conditioned medium from fibrin-stimulated BV-2 cells showed a significant reduction in total cell number compared with control conditioned media, consistent with reduced cell retention and/or increased cell loss. (**D**) Quantification of SOX2^+^CC3^+^ double-positive cells (per 100,000 SOX2^+^ cells). Conditioned medium from fibrin-stimulated BV-2 cells significantly increased SOX2^+^CC3^+^ double-positive labeling relative to control. All data were compared using an unpaired, two-tailed t test after confirming no significant difference in variance by F test. Circles represent individual wells (technical replicates); Control: n = 6–7, Fibrin: n = 7. All data are represented as mean ± SEM. Scale bar: 250 μm. White arrows indicate representative CC3^+^ cells or clusters of CC3^+^ cells; yellow arrows indicate SOX2^+^CC3^+^ double-positive cells; open arrows indicate areas where Hoechst nuclei are absent, but CC3^+^ signal is present.

We next tested whether this conditioned media could alter NPC survival *in vitro* (Fig. 5). NPCs exposed to conditioned medium from fibrin-stimulated BV-2 cells showed increased apoptotic signaling, reflected by a higher proportion of cleaved caspase 3-positive (CC3^+^) cells normalized to Hoechst^+^ nuclei (Fig. 5A white arrows, **&** 5B). We also observed a considerable overall reduction in Hoechst^+^ cell density per mm^2^ (Fig. 5A open arrows, **& 5C**). Because apoptosis in adherent neural cultures can be accompanied by loss of adhesion and detachment from the culture surface^101–104^, the reduced Hoechst^+^ cell density may reflect apoptosis-associated cell loss. Consistent with this, conditioned medium from fibrin-stimulated BV-2 cells also increased the number of SOX2^+^CC3^+^ double-positive cells (normalized to total SOX2^+^ cells), indicating that apoptotic signaling was elevated within the SOX2^+^ progenitor population (Fig. 5A yellow arrows, **&** 5D).

Together, these findings suggest that soluble factors generated by fibrin-stimulated BV-2 cells are capable of inducing NPC apoptosis and reducing NPC retention/survival *in vitro*. However, the specific soluble mediator or combination of mediators responsible for this effect remains unclear.

## 4. Discussion

Over the past three decades, interest in the molecular and cellular mechanisms linking maternal infection to altered brain developmental trajectories has grown steadily^105,106^. This topic is of clear public health importance, given the prevalence and societal burden of NDDs^8,107^. Within this framework, IAV-induced MIA has been proposed as a contributor to adverse neurodevelopmental outcomes through exaggerated maternal inflammatory milieu, although this remains an active and rapidly evolving area of investigation.

In this study, X31 infection elicited clear clinical signs of a maternal anti-viral pro-inflammatory response, most notably impaired maternal weight gain, consistent with our previous work^9,10,108^. This was accompanied by reduced fetal weight, shorter crown-to-rump length, and lower placental weight, indicating that maternal influenza exposure adversely affects fetoplacental development. These findings are consistent with intrauterine growth restriction and support the idea that maternal viral infection may disrupt the intrauterine environment in ways that broadly compromise fetal health outcomes^109–111^.

Given the well-established male bias in susceptibility to NDDs^112–114^, we examined whether the alterations identified in our model were influenced by fetal sex. We first focused on the SVZ, a major neurodevelopmental niche in the fetal brain^115–117^. The SVZ contains neural progenitor populations essential for brain development, and its dynamic cellular environment must be tightly regulated to support appropriate proliferative and differentiation programs^118–120^. In addition, the close association between SVZ progenitors and the local vasculature, together with evidence that periventricular vessels acquire mature BBB properties relatively late during development^121^, may make this region particularly sensitive to bloodborne inflammatory signals during MIA. The increased fibrinogen accumulation observed in the SVZ following maternal IAV exposure is consistent with our previous report^9^, where we also observed reduced abundance of endothelial tight junction CLDN5 in this region. This supports a localized alteration in blood-brain and blood-cerebrospinal fluid barrier permeability. Rather than reflecting a global disruption of fetal brain barrier integrity, this regional pattern suggests that maternal IAV exposure may selectively affect neurodevelopmental interfaces in which barrier properties are still undergoing maturation. In the SVZ, such increased permeability may reflect inflammation-induced interference with endothelial maturation and tight junction stability, as well as altered pericyte-endothelial coupling or regulated macromolecular transport. This interpretation is consistent with work from Bonnin and colleagues^122^ demonstrating that gestational MIA disrupts fetal BBB formation through COX2-dependent fetal microglial responses, impaired pericyte-endothelial interactions, and persistent BBB dysfunction in offspring. We further show that fibrinogen accumulation was accompanied by enhanced colocalization with Iba1^+^ microglia/macrophages within the SVZ, indicating that resident myeloid cells are recruited to the site of fibrinogen deposition.

This observation prompted us to ask whether similar patterns might also be present beyond the SVZ, in other neurodevelopmentally relevant regions. Accordingly, we extended our analysis to the Thalamus-3V interface and, more broadly, to the whole hemisphere. The thalamus was of particular interest given its central role in the maturation and integration of thalamocortical circuitry during development^123–125^. Prior work in a poly(I:C)-induced MIA model showed that the thalamus was reduced in size and exhibited weaker functional connectivity, particularly with cortical regions, together with greater segregation from broader brain networks^126^. These thalamocortical alterations may also be relevant for behavioral abnormalities previously described in poly(I:C)-MIA offspring^127,128^ and are consistent with evidence of thalamocortical dysfunction in SZ^129–131^.

Here, the increased fibrinogen-microglia colocalization observed across additional brain regions suggests that maternal IAV-induced vascular permeability extends beyond a single neurodevelopmentally relevant niche. This broader regional distribution may, in turn, have implications for the maturation and functional integration of circuits that are important for later brain function. Notably, this pattern was not modified by sex; male and female fetuses were equally susceptible to fibrinogen leakage following maternal IAV exposure.

Given the capacity of fibrinogen to engage microglia and promote inflammatory signaling under pathological conditions in mature animals^44,47,67,68,132–136^, we next asked whether fibrinogen accumulation was similarly accompanied by changes in markers related to microglial oxidative potential. We focused on p47^phox^, a key regulatory subunit required for stimulus-dependent NOX2 assembly and activation^86,137^, as an initial readout relevant to ROS-associated mechanisms in the developing brain. It is worth noting that redox status is maintained as a tightly regulated equilibrium and should not be viewed as inherently detrimental. Rather, moderate, physiological levels of ROS appear to be required for normal neurodevelopment^138^. In primary hippocampal neurons isolated from E18.5 rat embryos, disruption of NOX function—either through expression of a dominant-negative p22^phox^ construct or by inhibiting p47^phox^ translocation to the plasma membrane—delayed neuronal polarity acquisition and reduced axonal growth^139,140^. Mechanistically, these effects were linked to altered actin cytoskeleton dynamics and reduced Rac1 and Cdc42 activity^141,142^, supporting the idea that appropriate NOX-dependent redox signaling contributes to normal developmental programs. At the same time, disruption of this balance under inflammatory conditions may favor pathological responses^143,144^. NOX1 upregulation in fetal brains from poly(I:C)-challenged dams was associated with adverse neurodevelopmental outcomes, including Purkinje cell loss and autism-like behavioral abnormalities^145^. Using an LPS-induced MIA model, others have also demonstrated persistent neuroinflammatory and oxidative alterations in the offspring brain, together with synaptic deficits, supporting the idea that imbalanced redox signaling may contribute broadly to MIA-associated neuropathology^146^. This view is also consistent with reports of redox imbalance in the brains of autistic individuals^147,148^.

The increase in fibrinogen-associated Iba1^+^ cells we observed following maternal IAV infection raised the possibility that microglia/macrophages in the developing brain may likewise shift toward a more oxidation-prone phenotype. Consistent with this possibility, the increased density of Iba1^+^/p47^phox+^ cells in the whole hemisphere, Thalamus-3V, and SVZ, despite the absence of clear sex differences in p47^phox^ analyses, indicates that maternal IAV exposure is associated with a region-dependent increase in microglia/macrophage-lineage cells with p47^phox^ associated redox potential. Our *in vitro* data provides an important functional complement by showing that fibrin was sufficient to increase DHE oxidation in BV-2 microglial-like cells. While overall p47^phox^ signal was not impacted by maternal IAV infection, it should be noted that some p47^phox^ immunoreactivity was evident outside of Iba1^+^ profiles, indicating that Iba1^−^ cell populations may also contribute to NOX2-associated redox signaling in the fetal brain. Candidate contributors include neurons^139,140^ and vascular endothelial cells^149^ or pericytes^150^, although additional colocalization studies would be required to define these sources.

Given that redox-sensitive pathways are known to influence neural progenitor biology^98,151^, we next examined whether maternal flu altered NPC density in the fetal brain. Unlike some prior MIA models in which neural precursor populations are altered^152–158^, maternal IAV infection did not produce detectable changes in SOX2^+^ progenitor density at E16.5, indicating that the overall NPC population remains relatively stable at this stage. However, this does not exclude the possibility of more selective effects on specific progenitor subsets, as SOX2 is not sufficient to fully define neural progenitor identity or maturation state. We next asked whether maternal flu was associated with increased apoptotic vulnerability within SOX2^+^ progenitors and other fetal brain cell populations.

Apoptosis assessed by ApopTag revealed a sex-dependent treatment effect, with males showing greater vulnerability. This male-selective increase in apoptotic burden was particularly evident within the SOX2^+^ progenitor population across neurodevelopmentally relevant regions, including the Thalamus-3V interface and the SVZ. Importantly, the apoptotic response extended beyond SOX2^+^ progenitors, as SOX2^−^NeuN^−^ cells also exhibited a male-biased increase in cell death. This SOX2^−^NeuN^−^ population should be interpreted cautiously, as it likely contains multiple cell states rather than a single discrete lineage. These cells may include intermediate or glial-lineage progenitors, as well as immature neuronal/postmitotic cells that are no longer SOX2^+^ but have not yet reached a NeuN^+^ differentiated state^159,160^. This broader cellular involvement suggests that maternal flu may impact more than one neural/glial lineage, pointing to a wider disruption of the fetal neurodevelopmental environment in males. This pattern is broadly consistent with prior evidence suggesting that male offspring show greater susceptibility to adverse neurodevelopmental consequences following prenatal inflammatory challenge^161,162^, although the magnitude and nature of these effects vary across models^99,152,163,164^.

Importantly, the absence of a detectable reduction in SOX2^+^ progenitor cell density does not necessarily conflict with the increased apoptotic labeling observed within the SOX2^+^ population. These measurements likely capture different aspects of progenitor biology at a single developmental stage. While SOX2^+^ cell density reflects the overall size of the progenitor pool at E16.5, ApopTag labeling identifies cells actively undergoing DNA fragmentation at the time of assessment. Therefore, one possibility is that a subset of SOX2^+^ progenitors had already initiated apoptotic programs at or prior to E16.5, when apoptotic cells are still expected to be present as part of normal embryonic cortical development^165,166^, and that they retained SOX2 expression^167,168^. Under this scenario, these cells would still be counted as part of the SOX2^+^ population, even though they were already committed to cell death. Thus, maternal IAV infection may increase apoptotic vulnerability within the male progenitor compartment before producing an overt change in total SOX2^+^ cell density. Alternatively, this pattern may reflect a transient or regionally restricted injury response that is not sufficient to alter overall progenitor abundance at E16.5. Distinguishing between these possibilities would require assessment across additional developmental stages.

Notably, there were no sex or treatment differences in the number of postmitotic NeuN^+^ cells undergoing apoptosis, indicating that differentiated neurons are relatively protected. Additionally, while maternal flu did increase the abundance of Iba1^+^ cells colocalizing with fibrinogen, this was not sex specific, and thus it is unlikely that the observed male-biased apoptosis could be explained solely by increased BBB permeability and myeloid cell reactivity. However, it should be noted that male microglia differ in number, morphology, function, and maturational timelines compared to females^169–171^, and that male-biased neurodevelopmental outcomes have been consistently reported in other MIA models^172–175^. Thus, we cannot rule out the possibility that intrinsic features of the male brain, including microglial immune reactivity, may be driving the observed sex differences in overall apoptosis. Moreover, because inflammatory mediators (other than p47^phox^ signal) were not assessed in male and female fetal brains, we cannot determine whether this male-specific response reflects greater inflammatory exposure in males or greater sensitivity of male progenitors to the same maternal challenge.

Overall, these data suggest that a preserved overall SOX2^+^ density at this time point does not exclude the possibility of altered progenitor survival at later time points, particularly in males. Although postnatal compensatory mechanisms may partially restore progenitor populations, apoptosis during an active neurogenic window could also remove lineage-committed cells that cannot be fully replaced later. Future longitudinal studies are required to determine whether the observed increase in SOX2-positive and - negative apoptotic cells in males reflects a permanent or transient loss of specific neuronal or glial populations.

Importantly, prior work has shown that fibrinogen can directly influence adult SVZ-derived NPC fate by promoting astroglial differentiation and suppressing neuronal differentiation under specific *in vitro* conditions^176^. The same study also reported *reduced* apoptosis in fibrinogen-exposed adult NPC cultures. In contrast, our experiments were designed to test a distinct, indirect mechanism in which fibrin exposure first shifts microglia into a pro-oxidative state, thereby altering the soluble signals encountered by nearby progenitor cells. Rather than exposing NPCs directly to fibrinogen, we exposed them to conditioned medium from fibrin-stimulated BV-2 microglia-like cells. Conditioned medium was sufficient to increase NPC apoptosis, including an increase in the SOX2^+^CC3^+^ double-positive population, suggesting that fibrin-mediated microglial responses can generate soluble cues that negatively affect progenitor cell survival. These findings suggest that fibrin-related effects on NPC biology are highly context-dependent and may differ according to developmental stage, cellular environment, direct versus indirect exposure, and the specific endpoint analyzed.

It should be noted that these *in vitro* experiments relied on BV-2 microglia-like cells rather than primary microglia, and therefore should be interpreted as more of a reductionist model of fibrin-induced microglial responses. Future studies using primary microglia, ideally derived from developmentally relevant stages, will be necessary to confirm whether similar fibrin-induced secretory and redox responses occur in a more physiologically relevant context. In addition, we were constrained by the properties of each cell line and therefore were unable to assess sex as a biological variable; BV-2 cells are known to be genetically female^177,178^, whereas cortical NPCs were derived from mixed gender E15–18 embryos. Thus, we cannot determine whether fibrin-induced microglial responses or conditioned medium-induced NPC apoptosis may differ between male- and female-derived cells.

Because conditioned medium from fibrin-stimulated BV-2 microglia-like cells was sufficient to increase NPC apoptosis, we considered whether this effect could be associated with changes in the soluble cellular environment. Although most fibrin-responsive candidates were modestly decreased, the two increased factors, G-CSF and ICAM-1, may be relevant to neuroimmune and injury-associated signaling. G-CSF has been linked to neuroimmune signaling, neural progenitor responses, and protection against programmed cell death^179,180^. ICAM-1, in turn, is commonly associated with inflammatory activation^181^, and fibrinogen has been linked to ICAM-1-dependent inflammatory adhesion mechanisms^182^. However, because the array (which contained a defined panel of only 40 soluble immune mediators) revealed a mixed profile that included decreased chemokines, myeloid-associated trophic factors, tissue-remodeling mediators, and counter-regulatory inflammatory proteins, these data do not support a single clearly pro- or anti-inflammatory soluble signature. Interestingly, and in line with our hypothesis, fibrin stimulation significantly increased ROS in BV-2 microglia-like cells. While this finding supports oxidative stress as part of the BV-2 response to fibrin stimulation, it does not establish ROS as the direct transferable mediator responsible for NPC apoptosis. Given the short-lived nature of many highly reactive ROS species^183^, persistence of BV-2-derived ROS in conditioned media is unlikely to fully explain NPC death. However, more stable redox-related mediators^184^ or secondary oxidative products could still contribute to the conditioned environment.

Thus, while our findings indicate that fibrin exposure alters the BV-2 secretome, the functional relevance of these changes remains uncertain and the specific mediator(s) responsible for NPC apoptosis are not fully resolved. Future studies are needed to identify which fibrin-induced BV-2-derived factor, or combination of factors, mediates the observed effects on NPC apoptosis and survival.

Taken together, our data suggest that fibrinogen/fibrin-related effects on progenitor survival are shaped in-part by microglia/macrophage-mediated changes in the soluble microenvironment. To our knowledge, these findings provide the first evidence linking fibrinogen/fibrin accumulation, microglia-associated oxidative responses, and progenitor apoptosis in the fetal brain following maternal inflammatory challenge. Oxidative stress emerges as an important component of the fibrin-induced response, and, while unlikely to be the sole mediator of NPC apoptosis, may broadly direct survival and differentiation outcomes in neurogenic niches. Importantly, fibrinogen/fibrin accumulation in the developing SVZ during maternal IAV infection is consistent with increased neurovascular permeability, raising the possibility that additional circulating bloodborne factors may also access neurodevelopmentally sensitive regions during MIA. In this context, altered neurovascular permeability may not simply represent a downstream consequence of prenatal inflammation, but could also provide a route through which local pro-inflammatory signaling is amplified or sustained. It is also noteworthy that some effects of maternal IAV infection were sex-dependent, including male-biased apoptosis, whereas others were evident in both sexes, including fibrinogen accumulation and the Iba1^+^ NOX2-associated signature. Whether permeability dynamics, redox balance, and neurogenic potential in the offspring brain are restored through compensatory mechanisms, and whether sex-specific divergence continues over time, remains to be determined. Still, our observations indicate that myeloid-associated oxidative responses following fibrinogen/fibrin deposition may represent a previously unrecognized mechanism through which maternal inflammation contributes to apoptosis within neurogenic populations, with potential consequences for neurodevelopmental trajectories and NDD-relevant neuropathology.

## Supplementary Material

Supplementary Files

This is a list of supplementary files associated with this preprint. Click to download.

- SupplementalMaterialMIAReportingGuidelinesChecklist.pdf
- SupplementaryFiguresTables.pdf

## Data Availability

All data supporting the findings of this study are available within the paper and its Supplementary Information.
